# Craft Beer Produced by Immobilized Yeast Cells with the Addition of Grape Pomace Seed Powder: Physico-Chemical Characterization and Antioxidant Properties

**DOI:** 10.3390/foods13172801

**Published:** 2024-09-03

**Authors:** Danijel D. Milinčić, Ana S. Salević Jelić, Steva M. Lević, Nemanja S. Stanisavljević, Teodor Milošević, Vladimir B. Pavlović, Uroš M. Gašić, Nataša S. Obradović, Viktor A. Nedović, Mirjana B. Pešić

**Affiliations:** 1 Labororatory of Food Chemistry and Biochemistry, Faculty of Agriculture, University of Belgrade, Nemanjina 6, 11081 Belgrade, Serbia; danijel.milincic@agrif.bg.ac.rs; 2 Laboratory of Food Biotechnology, Faculty of Agriculture, University of Belgrade, Nemanjina 6, 11081 Belgrade, Serbia; ana.salevic@agrif.bg.ac.rs (A.S.S.J.); slevic@agrif.bg.ac.rs (S.M.L.); teoze99@gmail.com (T.M.); 3 Institute of Molecular Genetics and Genetic Engineering, University of Belgrade, P.O. Box 23, 11010 Belgrade, Serbia; nstan86@gmail.com; 4 Department for Mathematics and Physics, Faculty of Agriculture, University of Belgrade, Nemanjina 6, 11080 Belgrade, Serbia; vlaver@agrif.bg.ac.rs; 5 Institute of Technical Sciences of Serbian Academy of Sciences and Arts, Knez Mihailova 35/IV, 11000 Belgrade, Serbia; 6 Department of Plant Physiology, Institute for Biological Research Siniša Stanković—National Institute of Serbia, University of Belgrade, Bulevar Despota Stefana 142, 11060 Belgrade, Serbia; uros.gasic@ibiss.bg.ac.rs; 7 Faculty of Technology and Metallurgy, University of Belgrade, Karnegijeva 4, 11120 Belgrade, Serbia; ntomovic@tmf.bg.ac.rs

**Keywords:** craft beer, grape pomace seeds, immobilized yeast cells, phenolic compounds, UHPLC Q-ToF MS, Ca-alginate beads, hop-derived α-acids

## Abstract

The aim of this study was to produce and to characterize craft beer fermented by immobilized yeast cells with the addition of Prokupac grape pomace seed powder (2.5% and 5%), to obtain a beer enriched with phenolic compounds and improved sensory characteristics. The immobilization of the yeast cells was performed by electrostatic extrusion, while the obtained calcium alginate beads were characterized by light and scanning electron microscopy. Phenolic and hop-derived bitter compounds in beer with or without grape pomace seed powder (GS) phenolics were identified using UHPLC Q-ToF MS. The results indicated that GS adjunct significantly shortened the fermentation process of wort and increased the content of phenolic compounds, especially ellagic acid, flavan-3-ols and pro(antho)cyanidins in the final products compared to the control beer. A total of twenty (iso)-α-acids and one prenylflavonoid were identified, although their levels were significantly lower in beers with GS phenolics compared to the control beer. Beers with GS phenolics showed good antioxidant properties as measured by the reduction of ferric ions (FRP) and the scavenging of ABTS^•+^ and DPPH^•^ radicals. The concentration of immobilized viable yeast cells was higher than 1 × 10^8^ CFU/g wet mass after each fermentation without destroying the beads, indicating that they can be reused for the repeated fermentation of wort. Beers produced with 5% GS added to the wort exhibited the best sensory properties (acidity, astringency, bitterness intensity, mouthfeel, aftertaste and taste), and highest overall acceptability by the panelists. The results showed that grape pomace seed powder present a promising adjunct for the production of innovative craft beer with good sensory properties and improved functionality.

## 1. Introduction

Beer is one of the oldest and most widely consumed low-alcohol beverages in the world, traditionally made from barley malt, water, hops and brewer’s yeast [1,2]. Although the brewing technology is well-known and standardized, beer styles vary, change and follow trends and consumers’ preferences. Due to global consumer demand for beers, there is an increasing number of microbreweries producing innovative, authentic and complex craft beers that are more flavorful than commercial beers [1,3,4]. Innovations in craft beer production include changes in wort production, variations in the brewer’s yeast used and the use of innovative raw materials in the brewing process, as well as the addition of various adjuncts [3,5,6]. The bibliometric analysis and mapping of beer and brewing publications has shown that past research mostly analyzed the brewing process, while recent publications (from 2008 to 2018) focus on consumer health and environmental sustainability [7].

Previous studies have shown that craft beer is a good source of minerals, vitamins and proteins, as well as phenolic compounds from hops, so its moderate consumption could have some health benefits [8,9,10]. However, a growing number of studies are investigating the use of non-cereal adjuncts in the production of novel beers that can potentially be labeled as “functional“ beers [1,2,11]. The aim of producing these beers is to obtain products with unique sensory properties that are high in phenolic compounds and/or other antioxidants [1]. So far, craft beers enriched with various medicinal herbs and plants [12,13,14,15,16], broccoli products [17], various fruits/or extracts [16,18,19,20,21], mushroom extract [22] and propolis [23], have been successfully produced. These craft beers enriched with phenolic compounds have shown improved antioxidant properties and unique sensory characteristics. Moreover, some of them have shown an improved stability and shelf life [16] and have anticancer, anti-obesity and antidiabetic activities, as well as immunoprotective and cardioprotective effects [1].

The winemaking process generates significant amounts of by-products (pomace, seeds, skins and stems), which serve as a valuable source of phenolic compounds (PCs) due to the incomplete extraction of phenolic compounds during vinification [24]. It is estimated that approximately 30–40% of the total phenolic compounds extracted from grapes, with the majority of extractable phenolics coming from the seeds, account for around 38–52% of the solid by-products [25]. Until now, grape seed extracts have been successfully integrated into various food products, including cheese [26], yogurt [27], ice cream [28], dry fermented sausages [29] and goat milk-based products [30]. As grape pomace seed powder, it is mainly used in bakery and confectionary products [31]. This incorporation has enhanced the antioxidant and nutritional properties of these foods. To our knowledge, no craft beer enriched with phenolics from grape pomace seed powder has yet been produced and analyzed. Recently, Gasiński, Kawa-Rygielska, Mikulski, Kłosowski and Głowacki [5] pointed out the possibility of the valorization of white grape pomace in brewing technology and obtained beer with improved antioxidant properties, a higher phenolic content and lower acetaldehyde content than the control beer. As grape pomace seeds are considered a rich source of phenolic acids, flavan-3-ols and procyanidins [32,33,34,35], they may be a promising adjunct in the formulation of functional craft beer with a pronounced astringent mouthfeel. Furthermore, the use of grape pomace seeds will contribute to global efforts to achieve sustainable food production and reduce environmental pollution.

For these new types of beer, special attention should be paid to the manner and timing of the addition of adjuncts during the brewing process (wort boiling, fermentation, maturation and packaging), as these influence the extraction and concentration of bioactive compounds in the beer [2]. In this context, wort boiling has been shown to be the most effective step in the extraction of phenolic compounds from various adjuncts [21]. In addition, craft beer styles are strongly influenced by the type of brewer’s yeast and the fermentation process. Several previous studies have reported the use of free-suspended yeast cells for wort fermentation, while immobilized yeast cells are only sporadically used on an industrial level due to engineering problems, an unbalanced beer flavor and high cost claims [36,37] On the other hand, the use of immobilized cells in beer production offers several advantages, such as a higher fermentation productivity, the improved stability of yeast cells and the easier recovery of cells for reuse [38,39]. To the best of our knowledge, the use of immobilized yeast cells in the production of craft beer enriched with various adjuncts and their influence on the sensory and functional properties of these beers have not yet been investigated.

Considering all aspects, the aim of this study was the production and characterization of craft beer enriched with phenolic compounds from Prokupac grape pomace seed powder using immobilized yeast cells. The application of grape pomace seed powder in brewing technology could be one of the innovative strategies to valorize winemaking by-products. To characterize the craft beers and immobilized yeast cells, this study includes microscopic, spectrophotometric and UHPLC Q-ToF MS analyses, as well as the monitoring of fermentation kinetics, sensory properties, antioxidant activity and cell viability. This study presents a unique and innovative approach to craft beer production by enriching it with phenolic compounds derived from grape pomace seed powder, a winemaking by-product. Additionally, the use of immobilized yeast cells can enhance fermentation productivity, combining sustainability and efficiency in the brewing process.

## 2. Material and Methods

### 2.1. Materials

Sodium alginate and calcium chloride dihydrate were supplied by Carl Roth (Carl Roth, Karlsruhe, Germany). The chemicals used for the preparation of the yeast growth medium and yeast enumeration were purchased from Sigma-Aldrich, St. Louis, MO, USA. Brewer’s yeast (*Saccharomyces cerevisiae*, Fermoale AY4, AEB Brewing, San Polo, Italy) was kindly provided by BIP Brewery, Belgrade, Serbia.

### 2.2. Preparation of Grape Pomace Seed Powder

**Figure 1:**
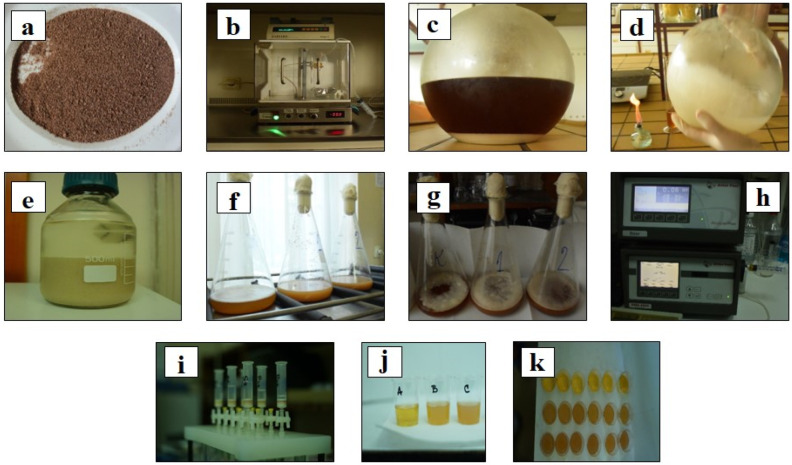
Scheme of the technological process of the production and analysis of craft beers without/with grape pomace seed powder: (**a**) Grape pomace seed powder. (**b**) Electrostatic extrusion device used for the immobilization of yeast cells. (**c**) Propagated yeast cells. (**d**) Yeast cells in carrier suspension. (**e**) Immobilized yeast cells in saline solution. (**f**) Inoculated hopped wort. (**g**) Fermentation of craft beer samples without and with grape pomace seed powder. (**h**) Alcolyzer Beer ME used for monitoring of fermentation kinetics. (**i**) Sample preparation for chromatographic analysis (solid–liquid extraction—SPE). (**j**,**k**) Beer samples for sensory analysis.

Grape pomace was obtained after fermentation of the indigenous variety Prokupac, from the winery “Vinska kuća Milinčić”, Aleksandrovac, Župa district, Serbia. The grape pomace seeds were manually separated from freshly pressed pomace, then finely ground in a coffee grinder (Bosch MKM 6003 UC, BSH Hausgeräte GmbH, Munich, Germany) and defatted with hexane by the immersion method (1:10 *w*/*v*), as previously described by Milinčić et al. [40]. Afterwards, the lipid fraction was separated, while the defatted seed powder was left in the chapel to evaporate the remaining hexane, additionally ground (Figure 1a) and used for the further production of craft beer.

### 2.3. Immobilization of Yeast Cells

For preparation of the pre-inoculum, 1 g of commercial yeast (*Saccharomyces cerevisiae*) was first hydrated in 40 mL of YPD liquid medium containing 1.5% (*w*/*v*) yeast extract, 2% peptone (*w*/*v*) and 2% dextrose (*w*/*v*) for 15 min at room temperature. After hydration, 300 mL of YPD was inoculated with the pre-inoculum to achieve an initial value of 0.2 OD at 600 nm. The inoculated medium was incubated at 30 °C for 24 h with continuous shaking at 200 rpm using an orbital shaker (Excella E24 Benchtop Incubator Shaker, New Brunswick Scientific, Edison, NJ, United States). After incubation, the yeast cell biomass was harvested by centrifugation at 4 °C and 2000× *g* for 20 min (refrigerated centrifuge 5804R, Eppendorf, Hamburg, Germany). The supernatant was discarded, and the biomass was washed with sterile water and centrifuged again under the same conditions. The prepared yeast biomass was weighed and used for encapsulation.

Before beer fermentation, the yeast cells were immobilized in calcium alginate beads. The yeast cells were immobilized by the electrostatic extrusion technique using encapsulation unit VAR V1 (Nisco Engineering AG, Zürich, Switzerland) (Figure 1b). The yeast cells (Figure 1c) were removed from the growth medium after sedimentation and mixed with a 1.5% (*w*/*v*) sodium alginate solution (Figure 1d) on a magnetic stirrer until a homogeneous cell suspension was obtained (Figure 1d). The cell suspension was extruded under the following conditions: the voltage was set at 7 kV, the distance between the needle tip and the gelling solution was 2.5 cm, the flow rate of the alginate/cells suspension was 90 mL/h (syringe pump 11 Harvard Apparatus, Holliston, MA, USA) and a blunt stainless steel needle with an inner diameter of 0.7 mm was used for extrusion. Calcium chloride solution (2%, *w*/*v*) was used as gelling solution. After a gelling time of 30 min, the calcium alginate (Ca-alginate) beads were removed from the gelling solution and washed with physiological saline solution (Figure 1e).

#### Morphological Properties and Microstructure of Immobilized Yeast Cells

The morphological properties of the Ca-alginate beads were investigated using a Leica DMLS light microscope (Leica, Wetzlar, Germany), equipped with a Leica-DC 300 camera (Leica, Germany) and Leica-IM 1000 software ver 4.0 (Leica, Germany). In addition, the microstructure of the Ca-alginate beads with immobilized yeast cells was investigated by scanning electron microscopy (SEM). For the preparation of the SEM analysis, the alginate beads were dehydrated with increasing ethanol concentrations in aqueous solutions (10, 20, 30, 40, 50, 60, 70, 80, 96% *v*/*v*) and twice with absolute ethanol (24 h for each dehydration step, at 4 °C). The dehydrated beads were stored in absolute ethanol at 4 °C in closed containers. A critical point carbon dioxide dryer (Critical Point Dryer, K850, Quorum Technologies, Laughton, UK) was used to remove the ethanol. To examine the internal structure, the dried alginate beads were carefully broken into two halves and attached to stubs with double-sided adhesive tape to expose the interior to be examined. The samples were coated with gold using a sputter coater (SCD 005, BAL-TEC), while SEM analysis was performed using a JEOL JSM-6390 microscope (JEOL, Peabody, MA, USA).

### 2.4. Brewing Technology

The hopped wort was obtained from the Dogma craft brewery, Belgrade, Serbia. Before fermentation, 0 g/100 mL (CBC), 2.5 g/100 mL (CB1) and 5.0 g/100 mL (CB2) of grape pomace seed powder were added to the hopped wort. The prepared wort/seed powder mixtures were autoclaved at 120 °C for 5 min. The sterilized wort samples were then cooled to 25 °C, transferred to simulated fermenters and inoculated with 10% immobilized yeast cells in a previously UV-sterilized laminar (Figure 1f,g). The simulated fermenters were placed in a cooled room with a temperature of 18 °C. During fermentation, the color of the samples was measured and the kinetics of fermentation were monitored. After the end of the primary fermentation, the produced craft beers were stored in the refrigerator at 4 °C for 24 h to settle and stabilize. The precipitated immobilized yeast cells were separated to determine the viability of the yeast cells after fermentation. The craft beers were then bottled in sterile bottles and stored in the refrigerator for further sensory analysis. In addition, samples of the craft beer were taken and immediately prepared for spectrophotometric and chromatographic analysis.

#### 2.4.1. Kinetics of Fermentation and Color Measurements

The kinetics of fermentation were monitored using the Alcolyzer Beer ME analysis system (DMA 4500, Anton Paar, Graz, Austria) (Figure 1h). The wort was sampled immediately after inoculation with the immobilized yeast cells and at regular intervals during fermentation under sterile conditions. The samples were degassed with ultrasound waves, filtered through cellulose filters, centrifuged at 17,000× *g* for 5 min and injected into the Alcolyzer Beer ME unit. The kinetics of fermentation were monitored over time by determining the alcohol content (%, *v*/*v*), original extract (°Plato), real extract (%, *w*/*w*), apparent extract (%, *w*/*w*) and apparent degree of fermentation (%, *w*/*w*). In addition to monitoring the fermentation kinetics, the wort samples were also analyzed for color using a spectrophotometric method [41]. For this purpose, the absorbance of the samples was measured at 430 nm using a UV–VIS spectrophotometer (HALO DB-20S, Dynamica Scientific Ltd., Livingston, UK). The color, expressed in EBC (European Brewing Convention) units, was calculated according to Equation (1):

(1) $$Color\;(EBC\;units)=A_{430}\times f\times 25$$

where *A*_430_ is the absorbance at 430 nm and *f* is the dilution factor.

#### 2.4.2. UHPLC Q-ToF MS Analysis of Bioactive Compounds

Priori to chromatographic analysis, the beer samples were passed through SPE cartridges, to concentrate the phenolic compounds and hop bitter compounds, as well as to remove residual sugars and other polar compounds. The SPE cartridges were conditioned with acidified methanol and milliQ water. Samples of degassed beer (10 mL) were then passed through SPE cartridges (Figure 1i) and washed with 5 mL of milliQ water, and finally the adsorbed compounds were eluted with 1.5 mL of acidified methanol (with 0.1% HCl). The prepared extracts were filtered through 0.22 µm filters and analyzed with an Agilent 1290 Infinity ultra-high-performance liquid chromatography (UHPLC) system coupled with a quadrupole time-of-flight mass spectrometer (6530C Q-ToF-MS from Agilent Technologies, Inc., Santa Clara, CA, USA), using the same method and operating parameters as previously described in [42]. For chromatographic separation, a Zorbax C18 column (2.1 × 50 mm, 1.8 µm) (Agilent Technologies, Inc., Santa Clara, CA, USA) and two mobile phases, ultra-purified water containing 0.1% HCOOH (A) and 98% acetonitrile containing 0.1% HCOOH (B), were used. The flow rate was set to 0.3 mL min^−1^, while the injection volume was 5 µL. The following gradient was used: 0–2 min (98% A), 2–17 min (98% A to 2% A), and in the next 5 min the gradient was returned to the initial condition (98% A) to restore column equilibrium. The Q-ToF MS is equipped with an Agilent Jet Stream electrospray ionization (ESI) source that operates in both positive and negative modes. An auto MS/MS acquisition mode (100–1700 *m*/*z*) was used for data acquisition, with the collision energy set to 30 eV. Agilent MassHunter software 10.0 was used for data analysis. Phenolic compounds and hop bitter compounds were identified based on their monoisotopic mass, MS fragmentation and the literature data [32,33,43,44,45,46,47,48,49]. The exact masses of the identified compounds were calculated using ChemDraw software (version 12.0, CambridgeSoft, Cambridge, MA, USA). The phenolic compounds were quantified using available standards (mg/100 mL). In the absence of specific standards, the content of some phenolic compounds was expressed in equivalents of the structurally closest (available) standard. Table S1 contains a list of phenolic compounds used for quantification, as well as their equation parameters, correlation coefficient (R^2^), linear range and LOQ. The percentage (%) of increase/decrease of individual hop acids in craft beers enriched with grape pomace seed phenolics (e.g., CB1 and CB2) was evaluated in comparison to the control craft beer (CBC), and calculated as a ratio of their areas.

#### 2.4.3. Total Phenolic Content and Antioxidant Properties

The total phenolic content (TPC), ferric reducing power (FRP), ABTS^•+^ scavenging activity (ABTS assay) and DPPH^•^ scavenging activity (DPPH assay) of the craft beers were determined using the methods described previously [32,50]. Prior to spectrophotometric analysis, the beer samples were degassed and filtered through 0.45 µm filters. Briefly, for the TPC determination, 35 µL of beer was mixed with the Folin–Ciocalteu reagent and 7.5% Na_2_CO_3_, and incubated for 1 h 30 min in the dark at room temperature before the absorbance was measured. To evaluate the ferric reducing power, 250 µL of beer, 250 µL of 0.2 M phosphate buffer and 250 µL of 1% potassium ferricyanide solution was mixed and incubated at 50 °C, for 20 min. Then, 10% TCA was added to the incubated mixtures, which were further vortexed and centrifuged at 17,000× *g* for 5 min (Sigma 201M Centrifuge, Osterode am Harz, Germany). The collected supernatants (135 µL) were mixed with milliQ water and 0.1% FeCl_3_, and incubated for 10 min before measuring the absorbance. For the DPPH assay, 40 µL of beer was mixed with 260 µL of a 0.1 mM working DPPH^•^ solution (DPPH dissolved in pure ethanol), and incubated for 20 min in the dark. For the ABTS assay, 15 µL of beer was mixed with a working solution of ABTS radical cations, and incubated for 7 min. For the spectrophotometric assays, absorbances were measured at 765 nm for TPC, 700 nm for FRP, 517 nm for DPPH^•^ and 734 nm for ABTS^•+^, using a UV–Vis microplate spectrophotometer (FlexA-200. Amtast, Lakeland, FL, USA). Results for TPC were expressed as mg gallic acid (GAE) equivalents (mg GAE/100 mL), while results for all the antioxidant assays were expressed as mg Trolox (TE) equivalents per 100 mL of beer (mg TE/100 mL).

#### 2.4.4. Cell Viability before and after Fermentation

The viability of the immobilized yeast cells before and after fermentation was determined using the spread plate method [51]. Briefly, 1 g of particles was mixed with 9 mL of 2% sterile tri-sodium citrate solution, and the prepared suspensions were mixed intensively until the particles dissolved completely. Decimal dilutions were prepared immediately after dissolving the particles in saline solution (NaCl 0.85%). Aliquots of the corresponding dilutions (0.1 mL) were added to previously prepared malt extract agar (MEA) plates—malt extract (20 g/L), glucose (20 g/L), peptone (1 g/L), agar (15 g/L), chloramphenicol (100 mg/L) and chlortetracycline (100 mg/L), pH 5.6—and spread evenly over the surface using sterile sticks. The plates were incubated at 25 °C for 5 days before the yeast colonies were counted. Only plates with a total number of 15 to 150 colonies were used for data analysis, and each diluted sample was plated and counted in triplicate. The number of CFU was expressed per g wet mass of alginate particles.

#### 2.4.5. Sensory Evaluation of the Craft Beers

The craft beers produced were sensory-evaluated using a quantitative descriptive analysis according to the previously described procedure [22] and the ISO standard [52]. All sensory evaluations with participants adhered to the Code of Professional Ethics of the University of Belgrade [53]. Participants provided informed consent before the evaluations, indicating their awareness of the confidentiality of their responses, their agreement to participate and allow their responses to be used, and their understanding that they could withdraw from the study at any time. They were also assured that no data would be released without their knowledge. The products tested were confirmed to be safe for consumption.

The sensory evaluation was carried out by a sensory panel comprised of 14 evaluators (6 female and 8 male evaluators) from the Faculty of Agriculture, University of Belgrade. All samples were tempered to 10 °C and served encoded in transparent plastic cups (40 mL) for sensory evaluation (Figure 1j,k). Water was served between beer samples for rinsing. Nine sensory parameters (color, odor, taste, aftertaste, mouthfeel, astringency, bitterness intensity, acidity, and overall acceptability) were evaluated using a five-point hedonic scale, with 1 and 5 points depicting a defective product and extremely desirable property, respectively. An importance factor (F = 0.05–0.5) was determined for each evaluated sensory parameter according to the MEBAK guidelines [54]. Thus, F = 0.05 was used for color, F = 0.29 for odor, aftertaste and acidity, F = 0.41 for taste, mouthfeel, astringency and bitterness acidity and F = 0.50 for overall acceptability. The points given to each parameter were multiplied by the corresponding importance factor, and the average value was calculated and presented.

### 2.5. Statistical Analysis

All results were performed in triplicate. Statistically significant differences between means were assessed using Tukey’s post hoc test (*p* < 0.05) (GraphPad Prism 6, San Diego, CA, USA). The graphs were prepared using GraphPad Prism 6 software (San Diego, CA, USA).

## 3. Results and Discussion

### 3.1. Morphological Properties of the Ca-Alginate Beads with Immobilized Yeast Cells

**Figure 2:**
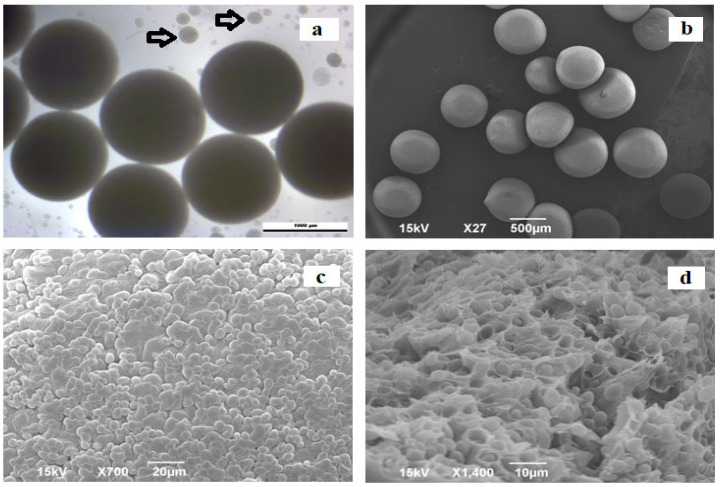
Images of the Ca-alginate beads loading yeast cells: (**a**) light microscopy image of the Ca-alginate beads (arrows indicate smaller “satellite” beads); (**b**) SEM images of the Ca-alginate beads, (**c**) their surface and (**d**) interior with visible immobilized cells.

The Ca-alginate beads with immobilized yeast cells (Figure 2a) had the shape of spherical particles with an average diameter of 1246.47 ± 43.12 μm. Smaller spherical beads, maintaining the size of the beads in a narrow range, were prepared by electrostatic extrusion. Smaller beads are preferable in fermentation processes considering nutrients’ limitations in larger-size carriers [55]. Figure 2a, marked by arrows, also shows smaller beads (with a diameter of less than 250 μm). These beads are known as “satellites” and are usually formed during the electrostatic extrusion of viscous solutions at higher voltages [56]. Since these small beads also contain yeast cells, they remain with the larger beads and thus contribute to fermentation. Critical point CO_2_ drying was employed for the Ca-alginate bead preparation for the SEM analysis. As can be observed in Figure 2b, the beads retained their spherical shape after drying. This indirectly indicates the good mechanical stability of the alginate network. The surface of the beads was affected by drying and the presence of yeast cells (Figure 2c). On the SEM micrographs of the beads’ interior (Figure 2d), yeast cells can be seen, entrapped inside the Ca-alginate matrix. This entrapment does not restrict yeast growth, and a high cell concentration can be achieved under optimal conditions [55]. The use of immobilized yeast cells could be a promising way to produce specific inoculums, especially in a dried form suitable for prolonged storage [57].

### 3.2. Fermentation Kinetics and Color Measurements

**Figure 3:**
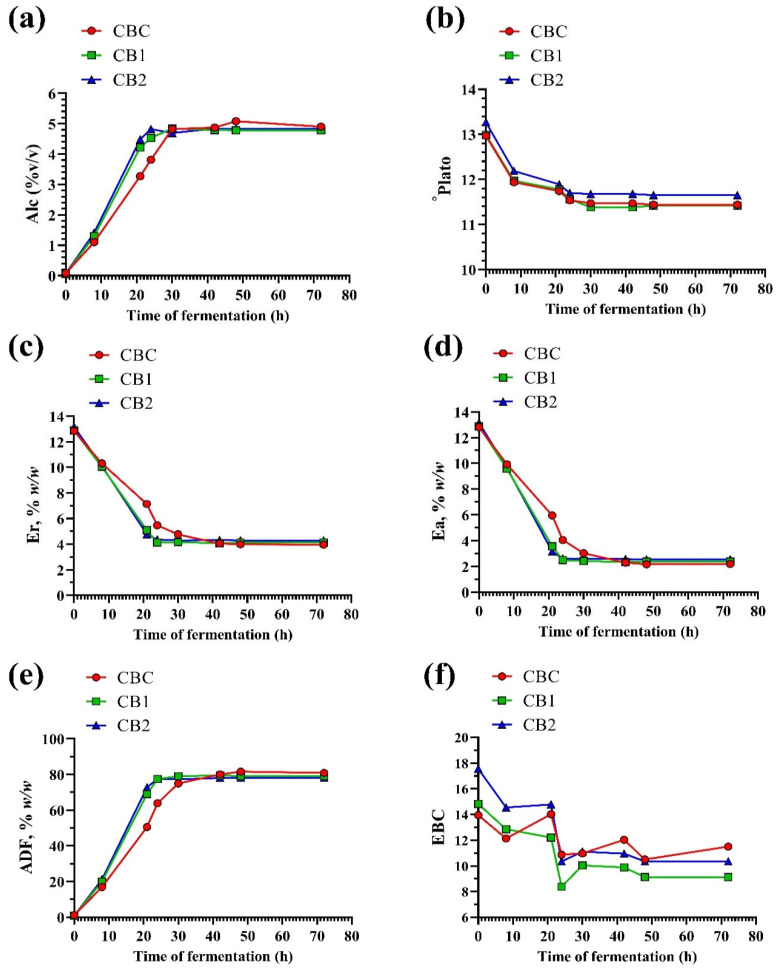
Fermentation kinetic monitoring: (**a**) Alcohol content, % *v*/*v*; (**b**) basic extract, Plato °; (**c**) real extract (Er), % *w*/*w*; (**d**) apparent extract (Ea), % *w*/*w*; (**e**) apparent degree of fermentation (ADF), % *w*/*w*; (**f**) color intensity (EBC). Abbreviations: CBC—craft beer control (0% grape pomace seed powder added to the wort); CB1—craft beer produced with 2.5% grape pomace seed powder added to the wort; CB2—craft beer produced with the 5.0% grape pomace seed powder added to the wort.

The quality parameters alcohol content, basic extract, real extract, apparent extract and apparent degree of fermentation were measured over a period of 80 h, to monitor the fermentation kinetics of the immobilized yeast cells of the control wort and the wort supplemented with 2.5% and 5% (*v*/*w*) GS (Figure 3). The measurement of these parameters showed that the added GS in the wort, regardless of the content, affects the fermentation kinetics within the first 30–42 h, depending on the parameter evaluated.

Within the first 30 h of fermentation, each wort sample showed an increase in alcohol content, with the increase being more pronounced in both samples supplemented with GS compared to the control sample. As expected, the initial basic extract was highest in the wort supplemented with 5% GS (*w*/*w*) and remained the highest throughout the fermentation period, likely due to the increase in total solids. The levels of real and apparent extract decreased in all samples during the first 30 h of fermentation, indicating the consumption of sugars, with the decrease being greater in the enriched wort samples than in the control wort, regardless of the content of the GS added. These results are supported by the values obtained for the apparent degree of fermentation, which show an increase in the fermentation rate during the first 30 h of monitoring and are more pronounced in the supplemented wort samples than in the control wort. The results obtained therefore revealed that the added GS positively affected the fermentation rate of the wort due to immobilized yeast cells. The higher fermentation efficiency of the wort supplemented with GS could be due to the increased content of fermentable sugars, nutrients and bioactive compounds that stimulate the yeast cells. For example, it was reported that supplementing the wort with banana juice had a positive effect on the viability and performance of brewer’s yeast cells due to the nutrients and sugars from the banana [58]. In the control wort, sugar consumption, reflected in the decrease in real and apparent extract and the increase in apparent degree of fermentation, continued until 42 h of fermentation, reaching values close to those of the supplemented wort samples within 30 h. Despite the described differences within this period of the fermentation process, all wort samples reached a plateau and similar values of the monitored quality parameters, i.e., alcohol content (~5%, *v*/*v*) (Figure 3a), basic extract (~11.5° Plato) (Figure 3b), real extract (~4.5%, *w*/*w*) (Figure 3c), apparent extract (~2.5%, *w*/*w*) (Figure 3d) and apparent degree of fermentation (~80%) (Figure 3e). The achieved steady state of the fermentation process and the achieved values of the monitored parameters were maintained until the end of fermentation. The levels of alcohol and real and apparent extracts in the produced craft beers correspond to the values reported for commercial pilsner beer [22]. This indicates the potential of using grape pomace seed powder (GS) as an adjunct in the production of craft beer.

Regarding the wort color, GS had the strongest effect after addition to the wort. As expected, the higher content of added GS resulted in a more intense wort color. The color varied across all wort samples during fermentation, but generally decreased (Figure 3f). Compared to the initial wort samples and the control wort, the color decreased more significantly at the end of fermentation in the samples supplemented with GS. Thus, the craft beer produced as a control without GS addition had the most intense color. Compared to some commercial beers, the color of the craft beers produced with immobilized yeast cells in our study is in the range of color values reported for European pale lager and Belgian strong pale ale [59].

### 3.3. UHPLC/Q-ToF/MS Analysis of Phenolic Compounds

The identification and quantification of phenolic compounds in the prepared craft beer without/with grape pomace seed phenolics were performed by UHPLC/Q-ToF/MS (Table 1). All phenolic compounds identified in the beer samples can be classified into three structurally different groups: (1) phenolic acid and derivatives (12 compounds), (2) flavan-3-ols (8 compounds), and (3) pro(antho)cyanidins (8 compounds). Most of the identified phenolic compounds originate from grape pomace seeds [32,33,35] and were confirmed in beer produced with GS. These phenolic compounds are probably largely extracted from the seed powder during the sterilization of the wort [2]. Moreover, the total content of all the identified phenolic compounds was significantly higher in beer enriched with GS phenolics, while their content increased with the increasing proportion of grape pomace seed powder (from 2.5% to 5%) in the wort. Thus, beer produced with 5% (*w*/*v*) GS added to the wort showed the highest content of quantified total phenolics (8169.47 µg/100 mL), then CB1 (6063.61 µg/100 mL), while the lowest content was confirmed in the control beer with only 955.26 µg/100 mL. Considering the results obtained, it can be concluded that the addition of 5% GS to the wort during beer production is optimal to achieving the maximum extraction and recovery of phenolic compounds from the grape pomace seed powder.

Among the phenolic acid derivatives, ellagic acid predominated in the beer produced with GS added to the wort, namely 450.30 µg/100 mL in CB1 and 604.76 µg/100 mL in CB2. This compound was extracted from grape pomace seeds, which is consistent with previous studies showing that a high level of ellagic acid is typical for Prokupac grape pomace seeds [32,33,50]. A high content of ethyl gallate was found in both CB1 and CB2 beer samples, probably due to the tendency of gallic acid to react with ethyl alcohol during beer fermentation. Dihydroxybenzoic acid was detected in all beer samples, but only quantified in CB1 and CB2 beer samples. Caffeoylquinic acid isomer I was the dominantly confirmed phenolic acid derivative in the control beer, but its presence in beer has been previously reported by other authors [15,46]. This phenolic acid ester was also quantified in CB1 beer, but it was not detected in CB2 beer. Other phenolic acids and their derivatives were not present or only present in trace amounts in the beer samples analyzed. Flavan-3-ols and their derivatives were predominant and accounted for more than 69% of the total quantified phenolics in beer samples with GS phenolics. Among them, the content of catechin was the highest (2110.50 µg/100mL for CB1 and 2776.98 µg/100 mL for CB2), followed by epicatechin, gallocatechin and chalcan-flavan-3-ol dimer isomers, while other detected flavan-3-ols were less abundant. Although these compounds are typical for grape pomace seeds [33], lower levels of catechin, epicatechin and gallocatechin were also detected in the control beer, probably derived from malt and hops [46,60]. Regarding proanthocyanidins, various isomers of the B-type procyanidin dimer, the B-type prodelphinidin dimer, the C-type procyanidin trimer and the B type procyanidin dimer gallate were detected and quantified, mainly in the CB1 and CB2 beer. In addition, the astringent sensation of beer with GS phenolics can be associated with the high content of flavan-3-ols and procyanidins, which are known to be carriers of astringency [61]. Among flavonols, only quercetin 3-*O*-(6″-*O*-rhamnosyl)-hexoside was detected, and it was present in traces in all the analyzed beer samples.

### 3.4. UHPLC Q-ToF MS Analysis of Hop-Derived Bitter Acids

Bitter acids (α-acids and β-acids) are characteristic compounds of beer that originate from hops and directly influence its organoleptic properties, impart bitterness and contribute to the foam stability of beer [46]. During wort boiling, the α-acids (n-, co- and ad- homologues) are converted into the more soluble iso-α-acids that have the same molecular mass [47] and are present as different *cis* and *trans* geometric isomers. The identification of hops-derived bitter acids was performed by UHPLC Q-ToF MS, taking into account the retention time, the exact *m*/*z* mass of the precursor ion and the typical MS fragments (Table 2). A total of 20 (*iso*)-α-acids were identified in the produced beer with/without GS phenolics. The majority of the identified bitter acids belong to the iso-α-acids, with typical fragments forming via dehydration and loss of side chains. Iso-α-acids show a typical fragment resulting from cleavage of the 4-methyl-pent-3-en-1-oxo side chain (-C_6_H_8_O, loss 96 *m*/*z*), while α-acids show a fragment resulting from loss of the prenyl chain (-C_5_H_9_, loss 69 *m*/*z*) [46]. On the other hand, *cis*/*trans* isomers and n/ad-forms of (*Iso*)-α- acids could not be distinguished, because they have an identical *m*/*z* mass of ions and the same MS fragmentation patterns; so, these isomers were considered together [43], as shown in Table 2. The molecular ions at *m*/*z* 331, *m*/*z* 333, *m*/*z* 347 and *m*/*z* 361 were tentatively recognized as *iso*-hulupone (compound **6**), *iso*-(*n*/*ad*)-posthumulone (compounds **7** and **8**), *iso*-cohumulone (compound **11**) and *iso*-(*n*/*ad*)-humulone (compounds **12, 13** and **14**), with typical fragments at 235 *m*/*z*, 237 *m*/*z*, 251 *m*/*z* and 265 *m*/*z* [M-H-C_6_H_8_O]^−^, respectively [48,49,62,63]. Compounds **17–19** were tentatively identified as isomers of (*n*/*ad*)-humulinone formed by the oxidation of iso-α-acids during the brewing process, while compound **20** was recognized as dihydro-*n*/*ad*-humulinone. The key fragments use to identify these compounds were 263 *m*/*z* (compounds **17–19**) and 265 *m*/*z* (compound **20**), obtained by loss of the 4-methyl-pent-3-en-1-oxo side chain and H_2_O ([M-H-C_6_H_8_O-H_2_O]^−^) [43,64]. Considering MS fragmentation, the only compounds identified as cohulupone (compound **3**), cohumulone (compound **10**) and dihydro cohumulinone (compound **15**) belonged to α-acids, as shown by their typical fragments resulting from the loss of the prenyl chain [64]. Typical fragments for the detection of cohumulone were at 278 *m*/*z* ([M-H-C_5_H_9_]^−^) and 235 *m*/*z* ([M-H-C_5_H_9_-C_3_H_7_]^−^) [65]. Compounds **2**, **4**, **5** and **9** were tentatively identified as derivatives of desoxy-tetrahydro-*n*/*ad*-humulone, desdimethyl-octahydro-iso-cohumulone, desoxy-iso-cohumulone⁄or hulupone-like and desdimethyl-*n*/*ad*- humulinone, respectively. These compounds were for the first time characterized and reported by Andrés-Iglesias, Blanco, Blanco and Montero [43] as metabolites for the differentiation of non-alcohol, low-alcoholic and regular beers. No β-acids were detected in the analyzed beer samples. These relatively insoluble acids were probably removed or oxidized during the brewing process [63]. The mutual comparison of the analyzed beers showed a significant decrease in the proportion of individually identified acids (except compound 4, Table 2) in beer enriched with GS phenolics compared to the control beer. Considering the results obtained and the fact that the hop-derived acids have a direct effect on the sensory properties of the prepared beer samples, it can be expected that beer produced with GS has a lower intensity of bitterness. In addition to bitter acids, hops also contain compounds from the prenylflavonoid subclass that have a basic flavonoid backbone linked to the prenyl group. Only one prenylflavonoid was detected in these samples, but it cannot be determined with certainty due to the lack of a standard. Three structurally similar compounds from the same subclass (desmethylxanthohumol, 6-prenylnaringenin, 8-prenylnaringenin) have the same exact mass (*m*/*z* 339), the same MS fragmentation patterns and typical fragments obtained by retro-Diels–Alder fragmentation [46,47].

### 3.5. Total Phenolic Content and Antioxidant Properties

**Figure 4:**
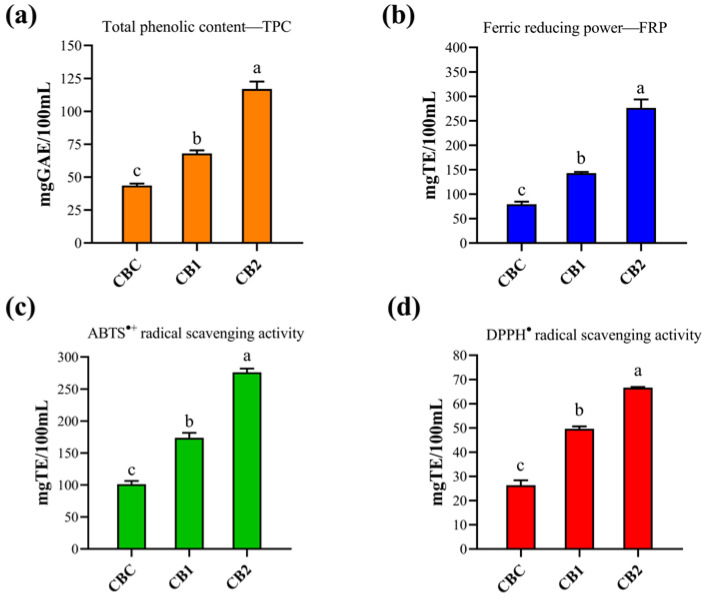
Spectrophotometric analysis: (**a**) Total phenolic content—TPC; (**b**) ferric reducing power—FRP; (**c**) ABTS^•+^ scavenging activity—ABTS assay; (**d**) DPPH^•^ scavenging activity—DPPH assay. Different lowercase letters indicate statistically significant differences according to the Tukey test (*p* < 0.05). Abbreviations: GAE—gallic acid equivalent; TE—Trolox equivalent. Samples: CBC—craft beer control (0% grape pomace seed powder added to the wort); CB1—craft beer produced with 2.5% grape pomace seed powder added to the wort; CB2—craft beer produced with 5.0% grape pomace seed powder added to the wort.

The results for the total phenolic content and antioxidant activity of the produced beers are shown in Figure 4a–d. The obtained values differed significantly (*p* < 0.05) among the analyzed beer samples for all applied spectrophotometric assays. The results of the Folin–Ciocalteu assay showed that the control beer had the lowest total PCs content (43.62 mgGAE/100 mL), while the TPC values of the beer samples enriched with GS phenolics were significantly higher, namely 67.98 mgGAE/100 mL (CB1) and 117.05 mgGAE/100 mL (CB2) (Figure 4a). These results were expected, due to the good reducing ability of phenolic compounds from grape pomace seeds, as previously reported by Milinčić et al. [33]. Although the TPC values were significantly higher, these spectrophotometric results showed the same increasing trend as the UHPLC Q-ToF MS results. The apparent higher TPC values in all the beer analyzed could be due to the presence of other non-phenolic compounds (proteins, amino acids, non-fermented sugars, some minerals or vitamins) that have a tendency to interact with the F-C reagent. Other studies have also shown that all beer formulated with fruits [18,19,20,21] or herbs [14] has a higher TPC content than the control beer. However, the TPC values for the enriched beer samples are the most common variable and highly dependent on the content of the adjunct extract/powder used and the brewing stages at which the adjunct is added. Finally, the TPC values obtained for beer with GS phenolics were higher than those of Gasiński, Kawa-Rygielska, Mikulski, Kłosowski and Głowacki [5] for beer made with white grape pomace (10 and 20% *w*/*w*), but lower than beer with grape skin extract (1, 5 and 10 mg/mL) [66].

All the prepared beer samples also showed a tendency to reduce Fe^3+^ ions (FRP), especially beer enriched with GS phenolics. The highest reduction potential was observed for CB2 (276.39 mgTE/100 mL), then for CB1 (143.19 mgTE/100 mL) and the lowest for the control beer (Figure 4b). As can be observed, increasing the content of added grape pomace seed powder to the wort (from 2.5% to 5%) has a direct effect on the diffusion/extraction of phenolic compounds, which have a good reducing ability. This assumption is supported by previous studies that showed a positive correlation between flavan-3-ols and procyanidins from grape pomace seed and ferric reducing power [24,33].

The evaluation of DPPH^•^ and ABTS^•+^ scavenging activities shows the ability of beer, i.e., its bioactive compounds, to neutralize free radicals. All the beer samples tested showed the ability to neutralize both DPPH^•^ and ABTS^•+^, while beer enriched with GS phenolics showed significantly better activity. In both assays, CB2 beer showed the best radical scavenging activity, i.e., 276.11 mgTE/100 mL for ABTS^•+^ and 66.63 mgTE/100 mL for DPPH^•^, while the control beer showed the lowest activity (Figure 4c,d). As can be observed, the beer samples have a better ability to scavenge ABTS^+^ than DPPH radicals, which is probably due to the hydrophobic nature of DPPH radicals and their selective reactivity with phenolic compounds. Comparison with the literature data for these two assays is limited, due to the different methodologies and the way the results are expressed. However, previous studies have also shown an improvement in ABTS^•+^ and DPPH^•^ scavenging activity for beer enriched with omija fruit [67], goji berry [21], white grape pomace [5], red grape skin extract [66] and jujube fruit [20].

### 3.6. Yeast Cell Viability after Fermentation

**Figure 5:**
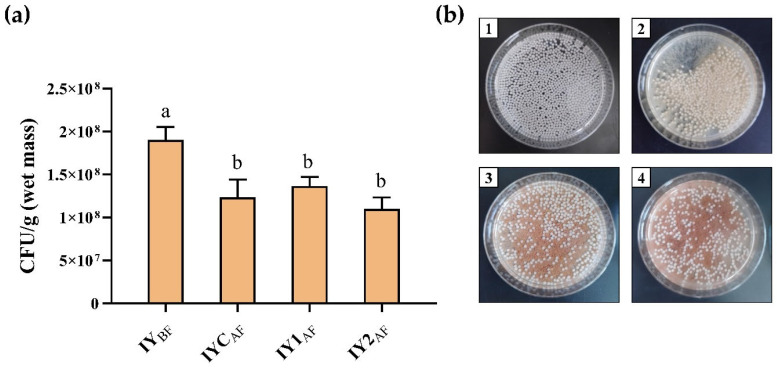
(**a**) Concentration of yeast cells before and after fermentation; (**b**) samples of alginate beads containing immobilized yeasts in different craft beers. Different lowercase letters indicate statistically significant differences according to the Tukey test (*p* < 0.05). Abbreviations: IY_BF_—immobilized yeast cell before fermentation (**b**) 1 IYC_AF_—yeast cell after fermentation of control craft beer (**b**) 2 IY1_AF_—yeast cell after fermentation of craft beer produced with 2.5% grape pomace seed powder added to the wort (**b**) 3 IY2_AF_—yeast cell after fermentation of craft beer produced with 5.0% grape pomace seed powder added to the wort (**b**) 4.

The viability of immobilized yeast cells before and after the fermentation of beer with/without GS was higher than 1 × 10^8^ CFU/g wet mass (Figure 5a,b). This indicates that the precipitated immobilized cells can potentially be reused for beer fermentation after fermentation. However, it should be emphasized that the concentration of yeast cells after fermentation decreased significantly compared to before fermentation. Nedović et al. [68] showed that the size of the alginate beads influences the concentration and growth of yeast cells. Smaller alginate beads (Figure 2a) probably “burst” during fermentation on first use, while the released yeast cells pass into the beer. The concentrations of yeast cells after fermentation in the beer samples did not significantly differ, indicating that the addition of grape pomace seed powder to the wort did not affect the viability of the immobilized yeast. However, while using of the same batch of immobilized yeast beads in repeated fermentations, we did not observe any disruption of the beads.

### 3.7. Sensory Properties of the Produced Craft Beers

**Figure 6:**
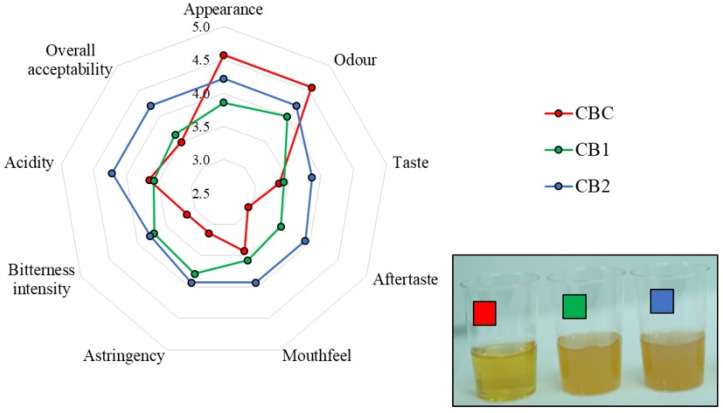
Sensory properties of control craft beer (CBC), craft beer produced with 2.5% grape pomace seed powder added to the wort (CB1) and craft beer produced with 5.0% grape pomace seed powder added to the wort (CB2).

The sensory quality properties of the craft beers obtained by fermentation with the immobilized yeast cells differed, depending on the addition and the quantity of grape pomace seed powder to the wort (Figure 6).

The control beer without GS phenolics was preferred by the sensory panelists in terms of appearance and odor to the beers produced from the GS-supplemented wort, which is reflected in the highest sensory scores. The beers produced from the GS-supplemented wort exhibited turbidity (Figure 6), which may be caused by the presence of grape pomace seed polysaccharides or interactions between the beer proteins and grape pomace seed polyphenols [16,22].

On the other hand, the GS addition to the wort resulted in beers with preferential taste properties compared to the control beer in terms of aftertaste, astringency and bitterness intensity, regardless of the added GS quantity (2.5 and 5%, *w*/*w*). This positive effect may be attributed to the flavan-3-ols of grape pomace seeds, including the various monomeric catechins and oligomeric or polymeric procyanidins, which are known to contribute to astringency and bitterness [6,69]. The perceived positive effect of the grape pomace seeds was also reflected in other taste properties evaluated in the beers produced. Namely, among the experimentally produced beers, the panelists rated the beer produced with 5% GS added to the wort as having the best taste, mouthfeel, and acidity. The effect of GS phenolics on the sensory preference for the resulting beers supports the ongoing trend in both industrial and craft beer production aimed at introducing new adjuncts to produce distinctive beers that are more sensorily complex and have a more intense flavor [70]. In addition, the supplementation of the wort with 5% GS resulted in the beer with the highest overall acceptability reported by the panelists. The improved taste properties and overall acceptability are important for the consumers’ perception of craft beers and subsequent purchasing decisions. The results obtained thus show the promising potential of grape pomace seeds, which are rich in phenolic compounds, for supplementing the wort before fermentation, with the aim of producing innovative beers characterized by sensory acceptance and improved functionality.

## 4. Conclusions

In this study, craft beer was produced with the addition of 2.5% and 5.0% of Prokupac grape pomace seed powder to the wort using immobilized yeast cells. The results indicate that the use of Ca-alginate-immobilized yeast cells in the production of craft beer enriched with GS phenolics can be successful because (1) the astringency and bitterness of the craft beer are improved; (2) the fermentation and production productivity are improved; (3) the reuse of yeast cells is enabled; and (4) the loss of phenolic compounds due to their interaction with the yeast cell walls is minimized.

The practical applications of these findings suggest that incorporating grape pomace seed powder into the brewing industry can offer a novel approach to sustainability by utilizing wine industry by-products, thereby contributing to waste reduction and resource efficiency. In addition, the use of immobilized yeast cells significantly shortens the fermentation process, impacting the efficacy of the process and saving time and energy. Furthermore, immobilized yeast cells can improve process sustainability by allowing for their reuse in multiple fermentation cycles, reducing the need for fresh yeast each time. This enriched craft beer presents an opportunity for brewers to produce unique products that meet consumer demand for functional beverages with health benefits.

However, this study identified some limitations. Sensory evaluation could be influenced by subjective differences among panelists, potentially affecting consistency. Although the interaction between phenolic compounds and yeast cell walls was minimized, further research is needed to quantify any remaining phenolic loss. Additionally, the reduced levels of key compounds, such as α-acids and prenylflavonoids in enriched beers, require further exploration to understand their influence on beer quality.

Regarding technological implementation, a craft brewery can be supplied with grape pomace seeds from a winery. The additional investment would be in equipment for grinding and defatting the grape seeds, which can be relatively easy to implement, though it may require some time and a moderate cost. However, for smaller craft breweries, this process could present more challenges in terms of space, time and cost compared to larger operations.

In conclusion, grape pomace seed powder is a promising adjunct for the production of innovative craft beer with improved sensory properties and functionality. However, future research should focus on addressing the identified challenges and exploring the health-promoting properties of such beers through additional in vitro and in vivo studies, opening doors for their commercialization in the functional beverage market.

## Figures and Tables

**Table 1:** Characterization and quantification of phenolic compounds in craft beer with and without grape pomace seed powder, using UHPLC Q-ToF MS. Target compounds, mean expected retention times (RT), molecular formula, calculated mass, *m*/*z* exact mass, mean mass accuracy (mDa) and MS fragments are presented.

RT	Compound Name	Formula	Calculated Mass	*m*/*z* Exact Mass	mDa	MS Fragments (%)	CBC (0%GS)	CB1 (2.5%GS)	CB2 (5.0% GS)
Phenolic acid and derivatives								**µg/100 mL**	
5.45	Hydroxybenzoic acid ^b^	C_7_H_5_O_3_^−^	137.02442	137.02178	2.64	107.01056 (2), **108.01857 (100)**, 109.02364 (13)	<LOQ	<LOQ	<LOQ
3.36	Dihydroxybenzoic acid (gentisic acid) ^a^	C_7_H_5_O_4_^−^	153.01880	153.01664	2.16	**108.01898 (100)**, 109.02631 (79)	<LOQ	92.23	106.65
1.55	Gallic acid ^a^	C_7_H_5_O_5_^−^	169.01370	169.01118	2.52	123.00633 (13), 124.01324 (77), **125.0212 (100)**, 126.02303 (5)	<LOQ	<LOQ	<LOQ
6.87	Caffeic acid ^a^	C_9_H_7_O_4_^−^	179.03440	179.03355	0.85	134.03343 (68), **135.04142 (100)**, 136.04448 (7)	<LOQ	<LOQ	<LOQ
7.71	Ethyl gallate ^b^	C_9_H_9_O_5_^−^	197.04500	197.04182	3.18	**124.01366 (100)**, 125.02059 (26), **169.01088 (2)**	-	36.58	47.74
4.24	Galloylglycerol ^b^	C_10_H_11_O_7_^−^	243.05103	243.04792	3.11	**124.01305 (100)**, 125.01936 (26), **168.00139 (1)**, 169.01007 (7)	-	<LOQ	<LOQ
8.15	Ellagic acid ^a^	C_14_H_5_O_8_^−^	300.99840	300.99661	1.79	145.02634 (9), 173.02153 (9), 185.02169 (11), 201.01519 (13), 229.01071 (20), 245.00621 (10), 257.00635 (8), 283.99209 (19), 299.9868 (19), **300.99392 (100)**	-	450.30	604.76
5.32	Vanilloloside ^b^	C_14_H_19_O_8_^−^	315.10800	315.10305	4.95	109.02781 (2), **123.04196 (100)**, 124.04444 (10), 153.05208 (57)	<LOQ	<LOQ	<LOQ
5.78	Caffeoylquinic acid is. I ^a^	C_16_H_17_O_9_^−^	353.08730	353.08623	1.07	134.03304 (5), **135.04205 (90)**, 136.04463 (11), 161.02029 (5), 173.0435 (3), 178.06898 (8), **179.03108 (40)**, **191.05243 (100)**	20.28	18.93	-
6.87	Caffeoylquinic acid is. II ^a^	C_16_H_17_O_9_^−^	353.08730	353.08920	−1.90	109.03236 (35), 111.04331 (38), 127.04667 (35), **135.0412 (100)**, 173.04221 (69), **179.03145 (49)**, 181.05164 (32), **191.05235 (67)**	<LOQ	<LOQ	<LOQ
4.78	Digalloyl hexoside is. I ^b^	C_20_H_19_O_14_^−^	483.07750	483.07412	3.38	**125.0209 (23)**, **169.01034 (100)**, 313.05237 (15), **331.06114 (17)**, 483.07152 (4)	-	<LOQ	<LOQ
5.99	Digalloyl hexoside is. II ^b^	C_20_H_19_O_14_^−^	483.07750	483.07603	1.47	124.01285 (13), **125.02154 (17)**, 168.00296 (7), **169.00982 (100)**, 170.01459 (8), 313.0512 (46), **331.06191 (18)**, 483.07265 (22)	-	<LOQ	<LOQ
∑							20.28	598.04	759.15
Flavan-3-ols and derivatives (carriers of astringency)									
6.67	Catechin ^a^	C_15_H_13_O_6_^−^	289.07120	289.06851	2.69	**109.02686 (95)**, **123.04219 (100)**, 125.02149 (43), 137.02151 (24), 151.0369 (31), 161.05632 (13), 187.03687 (10), 203.06718 (19), 221.07814 (12)	448.56	2110.50	2776.98
7.27	Epicatechin ^a^	C_15_H_13_O_6_^−^	289.07120	289.06898	2.22	**109.02615 (93)**, 121.02596 (27), **123.04157 (100)**, 125.02079 (41), 137.02084 (27), 151.03622 (31), 161.05521 (13), 203.06606 (18), 247.01958 (36)	91.63	817.92	954.21
4.31	Gallocatechin ^a^	C_15_H_13_O_7_^−^	305.06610	305.06197	4.13	109.02535 (17), 111.04193 (20), 124.01198 (15), **125.02097 (100)**, **137.02086 (34)**, 139.03679 (36), 165.01568 (14), **167.03209 (28)**, 219.06303 (7)	262.64	434.56	436.78
6.80	Epigallocatechin ^a^	C_15_H_13_O_7_^−^	305.06610	305.06540	0.70	109.02586 (78), 121.02668 (40), **125.02004 (100)**, **137.02051 (74)**, 161.01937 (63), 165.0125 (24), 177.05024 (20), **219.04178 (17)**	-	29.94	44.88
8.22	Epicatechin gallate ^a^	C_22_H_17_O_10_^−^	441.08220	441.08035	1.85	124.01275 (12), **125.021 (50)**, **169.01013 (100)**, 203.06594 (7), 245.07733 (14), **289.0667 (22)**, 290.07051 (4)	-	28.58	38.55
5.66	Epicatechin-hexoside ^c^	C_21_H_23_O_11_^−^	451.12400	451.12297	1.03	109.02619 (13), 125.02073 (16), 137.01963 (19), 165.01606 (12), 179.03194 (13), 203.06608 (19), **245.07746 (46)**, **289.06674 (100)**, 290.0686 (22)	-	70.54	85.82
6.67	Chalcan-flavan-3-ol dimer is. I ^c^	C_30_H_27_O_12_^−^	579.15030	579.14317	7.13	125.02129 (8), 137.02117 (7), 165.01593 (4), 179.0316 (8), 203.06795 (8), 205.04707 (9), 245.07829 (32), **289.06755 (100)**, 290.07123 (18)	<LOQ	457.61	688.91
7.27	Chalcan-flavan-3-ol dimer is. II ^c^	C_30_H_27_O_12_^−^	579.15030	579.14289	7.41	109.02562 (6), 123.04177 (3), 125.02111 (9), 137.02051 (7), 179.03031 (9), 203.06649 (9), 205.0457 (10), 245.07724 (32), **289.0667 (100)**, 290.06995 (19)	-	423.80	628.89
∑							802.83	4373.44	5655.03
*Pro (antho)cyanidins and derivatives (carriers of astringency)*									
6.45	Procyanidin dimer B type is. I ^d^	C_30_H_25_O_12_^−^	577.13460	577.13297	1.63	109.02687 (5), 125.0212 (8), 137.02169 (6), 179.03134 (8), 203.06758 (8), 205.04685 (8), **245.07818 (32)**, **289.06792 (100)**, 290.07144 (11)	31.54	157.70	286.08
7.07	Procyanidin dimer B type is. II ^d^	C_30_H_25_O_12_^−^	577.13460	577.13164	2.96	125.0209 (78), 137.02014 (11), 161.02007 (23), 205.04439 (7), **245.07585 (20)**, **289.06625 (100)**, 290.07029 (19), 339.08218 (10), **407.07089 (80)**, 425.08362 (7)	-	65.40	104.22
4.24	B type prodelphinidin dimer is. I ^d^	C_30_H_25_O_13_^−^	593.12959	593.12439	5.20	**125.02157 (69)**, 177.01565 (80), **245.07743 (16)**, 273.03602 (10), **289.06714 (85**), 339.08109 (12), **407.07203 (100)**, 425.07974 (10)	48.17	157.15	171.39
5.39	B type prodelphinidin dimer is. II ^d^	C_30_H_25_O_13_^−^	593.12959	593.12633	3.26	**125.02103 (73)**, 177.01556 (76), 245.07834 (18), 273.03567 (10), **289.06740 (84)**, 339.08302 (14), **407.07303 (100)**, 425.08266 (11)	52.44	103.98	113.24
7.68	B type procyanidin dimer gallate ^d^	C_37_H_29_O_16_^−^	729.14566	729.14313	2.53	**125.02093 (37)**, **169.01008 (21)**, 271.05517 (14), **289.0666 (80)**, 290.07017 (13), **407.07168 (100)**, 408.07294 (28), 441.07322 (10), 451.09645 (21), **577.12136 (13)**	-	44.73	79.36
6.53	B type procyanidin trimer is. I ^e^	C_45_H_37_O_18_^−^	865.19802	865.19536	2.66	**125.01991 (96)**, 243.0246 (29), **287.05074 (100)**, **289.06765 (57)**, **407.07436 (66)**, 425.08143 (59), 451.09605 (29), 575.11216 (55), **577.12083 (64)**, 865.18724 (48)	-	125.44	220.22
6.87	B type procyanidin trimer is. II ^e^	C_45_H_37_O_18_^−^	865.19802	865.19335	4.67	**125.0216 (100)**, 161.02182 (35), 245.04515 (31), **287.05295 (97)**, **289.06618 (86)**, **407.07193 (74)**, 425.08571 (57), 451.10024 (43), 575.11398 (40), **577.13152 (69)**, 695.13769 (46), 713.14353 (32), 865.19369 (35)	-	235.53	406.64
7.54	B type procyanidin trimer is. III ^e^	C_45_H_37_O_18_^−^	865.19802	865.19362	4.40	125.02128 (92), 161.01983 (27), 245.04142 (27), **287.04982 (100)**, **289.06626 (74)**, **407.07179 (69)**, 413.08225 (28), 425.07936 (50), 451.09678 (34), **577.12663 (52)**, 695.13371 (36), 713.14206 (23), 865.18764 (28)	-	202.19	374.13
∑							132.15	1092.13	1755.29
Flavonol derivatives									
8.02	*Quercetin 3-O-(6″-O-rhamnosyl)-hexoside* ^f^	C_27_H_29_O_16_^−^	609.14611	609.14524	0.86	150.99976 (5), 178.99516 (3), 271.0187 (8), **300.02243 (100)**, 301.02915 (77)	<LOQ	<LOQ	<LOQ
**∑∑**							955.26	6063.61	8169.47

Abbreviations: is.—isomer; <LOQ—less than limit of quantification; “-”—nonidentified compound; GS—grape pomace seed powder. Samples: CBC—craft beer control (0% grape pomace seed powder); CB1—craft beer with 2.5% grape pomace seed powder; CB2—craft beer with 5.0% grape pomace seed powder. Compound quantities expressed using available standards ^a^; compounds expressed as gallic acid equivalent ^b^; compounds expressed as epicatechin equivalent ^c^; compounds expressed as procyanidin B1 equivalent ^d^; compounds expressed as procyanidin C1 equivalent ^e^; compounds expressed as quercetin equivalent ^f^.

**Table 2:** Characterization and percentage (%) of hop (*H. Lupulus* L.)-derived bitter compounds in craft beer with and without grape pomace seed powder, using UHPLC Q-ToF MS. Target compounds, mean expected retention times (RT), molecular formula, calculated mass, *m*/*z* exact mass, mean mass accuracy (mDa) and MS fragments are presented.

No	RT	Compound Name	Formula	Calculated Mass	*m*/*z* Exact Mass	mDa	MS Fragments (%)	CBC (0%GS)	CB1 (2.5%GS)	CB2 (5.0%GS)
*(Iso)-α-acids*								% (% of lose)		
1	12.05	Hulupinic acid	C_15_H_19_O_4_^−^	263.12830	263.12433	3.97	123.00615 (12), **125.99276 (100)**, 126.99623 (7), 137.99271 (7), 139.00046 (10), **151.00134 (37)**, 165.05173 (6), **179.03146 (8)**, **193.04723 (15)**	100	109.7 (-)	80.9 (19.1)
2	13.48	Derivative of desoxy-tetrahydro-*n*/*ad*-humulone	C_18_H_33_O_4_^−^	313.23790	313.23473	3.17	127.07332 (11), 129.08882 (47), **183.13532 (100)**, 184.1387 (14), **195.13506 (6)**, 277.21219 (6), **295.22312 (6)**, 313.23347 (3)	100	52.7 (47.3)	62.2 (37.8)
3	12.46	Cohulupone	C_19_H_25_O_4_^−^	317.17530	317.17184	3.46	111.04264 (41), 133.06318 (42), **152.04504 (100)**, 153.04856 (11), **180.03998 (33)**, 193.04771 (8), **205.06588 (88)**, 206.07051 (14), 219.09951 (11), **220.10703 (36)**, 233.07867 (60), 234.08222 (10), **248.1022 (18)**	100	73.7 (26.3)	65.4 (34.6)
4	10.92	Desdimethyl-octahydro-*iso*-cohumulone	C_18_H_33_O_5_^−^	329.23280	329.22901	3.79	125.09433 (5), 127.11033 (13), 139.10989 (18), 171.09981 (42), 183.13602 (22), 193.11996 (8), **211.13085 (100)**, 212.13389 (17), **229.14098 (32)**, 230.14422 (5)	100	104.6 (-)	106.0 (-)
5	13.00	Desoxy-*iso*-cohumulone⁄or hulupone-like	C_20_H_27_O_4_^−^	331.19090	331.18728	3.62	125.05836 (34), **166.0606 (100)**, 167.05951 (13), 191.06802 (49), 194.05577 (32), 205.05262 (16), **219.07866 (80)**, 220.08251 (13), **234.123 (32)**, 247.09469 (59), 262.11782 (21)	100	66.4 (33.6)	55.9 (44.1)
6	14.41	*Iso*-Hulupone	C_20_H_27_O_4_^−^	331.19148	331.18754	3.95	123.00519 (47), **125.05746 (87)**, 164.08067 (24), 165.05239 (52), **166.05965 (100)**, 167.06488 (20), 191.06706 (75), 192.07463 (62), 221.11397 (19), **235.12962 (72)**	100	58.5 (41.5)	35.5 (64.5)
7	11.59	*Iso*- (*n*/*ad*)-posthumulone is. I	C_19_H_25_O_5_^−^	333.17020	333.16705	3.15	111.04256 (52), 167.06726 (10), 168.03977 (15), 169.04714 (21), 181.04757 (8), **209.11404 (4)**, **221.07836 (13)**, **237.10996 (100)**, 238.11351 (18)	100	33.9 (66.1)	19.8 (80.2)
8	13.21	*Iso*- (*n*/*ad*)-posthumulone is. II	C_19_H_25_O_5_^−^	333.17020	333.17041	−0.21	111.0061 (7), 125.05815 (11), 151.03733 (18), 163.07332 (25), **167.0319 (100)**, 168.0391 (62), 195.02632 (44), 219.09929 (37), 220.10455 (11), **223.09406 (7)**, **237.10959 (10)**	100	68.5 (31.5)	55.2 (44.8)
9	8.56	Desdimethyl-*n*/*ad*-humulinone	C_19_H_23_O_6_^−^	347.14950	347.14899	0.51	111.04198 (47), 125.0568 (10), 193.08256 (19), 231.06157 (14), 235.09467 (14), 259.09426 (6), 285.14461 (9), 305.1337 (8), **329.13289 (6)**, **347.14537 (100)**, 348.14809 (27)	100	128.0 (-)	91.9 (8.1)
10	16.84	Cohumulone	C_20_H_27_O_5_^−^	347.18580	347.18265	3.15	165.05258 (10), 166.0595 (4), 193.04719 (18), 207.06296 (53), **223.05777 (16)**, **235.0586 (100)**, 236.06274 (19), **278.11254 (80)**, 279.11606 (17)	100	65.7 (34.3)	69.9 (30.1)
11	14.89	*Iso*-cohumulone	C_20_H_27_O_5_^−^	347.18580	347.18310	2.70	111.04208 (31), 125.05762 (15), 165.05263 (16), **181.04694 (100)**, **182.05414 (87)**, 207.13539 (17), 209.04151 (53), **233.11433 (45)**, 234.1198 (14), **251.12487 (13)**	100	101.6 (-)	92.1 (7.9)
12	15.37	*Iso*- (*n*/*ad*)-humulone is. I	C_21_H_29_O_5_^−^	361.20150	361.19834	3.16	125.05838 (47), 167.06825 (11), 179.06852 (23), **195.06315 (92)**, **196.07057 (100)**, 197.07418 (13), 221.15171 (20), **223.0579 (58)**, 235.13084 (10), **247.13086 (42)**, 248.13594 (13), **265.14134 (14)**	100	75.0 (25.0)	43.2 (56.8)
13	15.57	*Iso*- (*n*/*ad*)-humulone is. II	C_21_H_29_O_5_^−^	361.20150	361.19905	2.45	125.05768 (46), 163.07306 (27), 167.06757 (11), 179.06772 (22), **195.06235 (100)**, 196.06967 (93), 221.15068 (18), **223.05695 (54)**, **247.13007 (47)**, 251.12469 (11), **259.12231 (10)**, **265.14019 (16)**	100	103.8 (-)	93.2 (6.8)
14	16.11	*Iso*- (*n*/*ad*)-humulone is. III	C_21_H_29_O_5_^−^	361.20150	361.19836	3.14	125.0582 (33), 163.07365 (19), 179.06825 (17), **195.06328 (100)**, 196.07059 (95), 221.15165 (17), 223.05838 (49), **247.13104 (44)**, 251.1256 (11), **265.14128 (15)**	100	62.5 (37.5)	52.1 (47.9)
15	13.54	Dihydro-cohumulinone	C_20_H_29_O_6_^−^	365.19640	365.19317	3.23	181.04691 (3), 195.06272 (8), 209.07799 (11), **223.058 (26)**, 237.07351 (50), 238.07798 (9), **296.12313 (100)**, 297.12638 (23)	100	90.8 (9.2)	93.8 (6.2)
16	11.32	(*Iso*)- (*n*/*ad*)-Prehumulone	C_21_H_27_O_6_^−^	375.18080	375.17985	0.95	125.05814 (17), 139.07366 (43), 191.07187 (7), 211.05809 (7), 220.07157 (7), **223.13034 (14)**, 239.05301 (8), **263.12551 (100)**, 264.12887 (15), 291.12044 (4)	100	59.8 (40.2)	43.5 (56.5)
17	11.45	(*n*/*ad*)-humulinone is. I	C_21_H_29_O_6_^−^	377.19640	377.19187	4.53	125.05807 (19), 139.07367 (47), 179.06844 (5), 192.07635 (7), 211.0581 (8), **220.07185 (9)**, **223.13093 (12)**, 239.05299 (9), **263.12553 (100)**, 264.12922 (22), 291.12029 (4)	100	75.6 (24.4)	66.4 (33.6)
18	11.86	(*n*/*ad*)-humulinone is. II	C_21_H_29_O_6_^−^	377.19640	377.19279	3.61	125.05817 (22), 139.07356 (25), 179.06802 (7), 195.06312 (15), 207.10217 (8), 211.0582 (7), **220.07235 (6)**, **223.1303 (15)**, 239.05285 (8), **263.12559 (100)**, 264.12824 (19), 283.15112 (6)	100	76.5 (23.5)	66.0 (34.0)
19	12.67	(*n*/*ad*)-humulinone is. III	C_21_H_29_O_6_^−^	377.19640	377.19287	3.53	125.0584 (21), **139.07372 (100)**, 141.05346 (14), 165.05261 (7), 179.06831 (7), 181.12033 (6), **223.1311 (20)**, **263.12545 (39)**, 264.12903 (7)	100	88.9 (11.1)	78.4 (21.6)
20	11.92	Dihydro-*n*/*ad*-humulinone	C_21_H_31_O_6_^−^	379.21210	379.20740	4.70	125.05746 (52), 127.0729 (29), 155.06825 (25), 179.06809 (33), 195.06172 (39), 207.09827 (89), **210.04955 (16)**, 237.10824 (27), 251.12092 (17), **265.13808 (100)**, 266.14463 (32), **283.14996 (54)**, 310.13721 (16), 321.16509 (26)	100	75.6 (24.4)	66.7 (33.3)
Prenylflavonoids										
21	14.01	Desmethylxanthohumol or 8-prenylnaringenin or 6-prenylnaringenin	C_20_H_19_O_5_^−^	339.12320	339.12051	2.69	**119.04678 (100)**, 120.05055 (10), **133.06233 (34)**, **151.07291 (15)**, **175.07185 (14)**, **219.06188 (50)**, **220.06562 (8)**, 245.07799 (3)	100	32.5 (67.5)	25.0 (75.0)

Abbreviations: is.—isomer; GS—grape pomace seed powder. Samples: CBC—craft beer control (0% grape pomace seed powder added to the wort); CB1—craft beer produced with 2.5% grape pomace seed powder added to the wort; CB2—craft beer produced with the 5.0% grape pomace seed powder added to the wort. Percentage (%) of increasing/decreasing individual hop acids in CB1 and CB2 beer compared to their content in control craft beer (CBC).

## Data Availability

The original contributions presented in the study are included in the article/Supplementary Materials, further inquiries can be directed to the corresponding authors.

## Supplementary Materials

The following supporting information can be downloaded at: https://www.mdpi.com/article/10.3390/foods13172801/s1, Table S1: Equation parameters and correlation coefficient (R^2^) of used phenolic standards for quantification.
