# Supplementary material for: Craft Beer Produced by Immobilized Yeast Cells with the Addition of Grape Pomace Seed Powder: Physico-Chemical Characterization and Antioxidant Properties
Source: Foods. 2024 Sep 3;13(17):2801. doi: 10.3390/foods13172801 (PMC11395119; doi:10.3390/foods13172801)
Supplement: Supplementary file 1 [file foods-13-02801-s001.zip › foods-3156432-supplementary.pdf]

Supplementary Materials:.

Table S1. Equation parameters and correlation coefficient (R^2) of used phenolic standards for quantification.

Standards	Y=a*X±b	R^2	Linear range (ppm)	LOQ (µg/mL)
Gentisic acid	Y=1726721*X+466061	0.9924	0.1-10	3.33
Galic acid	Y=4611996*X-637903	0.9964	0.1-10	2.58
Ellagic acid	Y=47569*X+18865	0.9941	0.1-10	2.68
Caffeic acid	Y=3049527*X+856403	0.9921	0.1-10	3.65
Chlorogenic acid	Y=1349366*X-134914	0.9970	0.1-9	1.88
Catechin	Y=120612*X+43508	0.9924	0.1-6	2.04
Epicatechin	Y=212084*X+42453	0.9907	0.1-5	1.93
Gallocatechin	Y=103169*X+1791	0.9966	0.1-10	2.02
Epigallocatechin	Y=182069*X+16006	0.9960	0.1-9	2.09
Epicatechin gallate	Y=1598366*X+270950	0.9900	0.1-4	1.83
Procyanidin B1	Y=972669*X-290745	0.9915	0.1-9	3.06
Procyanidin C1	Y=97466*X+11833	0.991	0.1-9	2.94
Quercetin	Y=769214*X+825343	0.9915	0.1-7	2.64
